# Supplementary material for: Transcriptome Analysis Reveals Vimentin-Induced Disruption of Cell–Cell Associations Augments Breast Cancer Cell Migration
Source: Cells. 2022 Dec 13;11(24):4035. doi: 10.3390/cells11244035 (PMC9776984; doi:10.3390/cells11244035)
Supplement: Supplementary file 1 [file cells-11-04035-s001.zip › DEGs and enrichment analysis.zip!/DEGs and enrichment analysis/WT_VIMvsVector GO enrichment/GSEA/WT_VIM/GLIAL_CELL_DEVELOPMENT/GLIAL_CELL_DEVELOPMENT(GO_0021782).html]

Details for gene set GLIAL\_CELL\_DEVELOPMENT(GO:0021782)[GSEA]


|  || Dataset | fpkm.sample |
| Phenotype | sample.cls |
| Upregulated in class | WT\_VIM |
| GeneSet | GLIAL\_CELL\_DEVELOPMENT(GO:0021782) |
| Enrichment Score (ES) | 0.9035958 |
| Normalized Enrichment Score (NES) | 1.4613835 |
| Nominal p-value | 0.0 |
| FDR q-value | 0.16299984 |
| FWER p-Value | 0.163 |
Table: GSEA Results Summary

  

Fig 1: Enrichment plot: GLIAL\_CELL\_DEVELOPMENT(GO:0021782)
  
Profile of the Running ES Score & Positions of GeneSet Members on the Rank Ordered List

  

| PROBE | DESCRIPTION   (from dataset) | GENE SYMBOL | GENE\_TITLE | RANK IN GENE LIST | RANK METRIC SCORE | RUNNING ES | CORE ENRICHMENT || 1 | ENSG00000084207 | GSTP1 |  |  | 175 | Infinity | -0.0030 | Yes |
| 2 | ENSG00000125820 | NKX2-2 |  |  | 225 | Infinity | -0.0038 | Yes |
| 3 | ENSG00000108381 | ASPA |  |  | 1258 | Infinity | -0.0213 | Yes |
| 4 | ENSG00000087250 | MT3 |  |  | 1710 | Infinity | -0.0289 | Yes |
| 5 | ENSG00000026025 | VIM |  |  | 2388 | 1709.539 | 0.8348 | Yes |
| 6 | ENSG00000139352 | ASCL1 |  |  | 2390 | 134.348 | 0.9036 | Yes |
| 7 | ENSG00000105695 | MAG |  |  | 2953 | 5.112 | 0.8967 | No |
| 8 | ENSG00000050555 | LAMC3 |  |  | 3283 | 4.008 | 0.8932 | No |
| 9 | ENSG00000186868 | MAPT |  |  | 3555 | 3.590 | 0.8904 | No |
| 10 | ENSG00000100146 | SOX10 |  |  | 3690 | 3.389 | 0.8899 | No |
| 11 | ENSG00000092621 | PHGDH |  |  | 3864 | 3.194 | 0.8886 | No |
| 12 | ENSG00000108797 | CNTNAP1 |  |  | 3892 | 3.146 | 0.8897 | No |
| 13 | ENSG00000149256 | TENM4 |  |  | 3916 | 3.117 | 0.8910 | No |
| 14 | ENSG00000169071 | ROR2 |  |  | 3924 | 3.097 | 0.8924 | No |
| 15 | ENSG00000184486 | POU3F2 |  |  | 4174 | 2.884 | 0.8897 | No |
| 16 | ENSG00000146216 | TTBK1 |  |  | 4279 | 2.799 | 0.8893 | No |
| 17 | ENSG00000142208 | AKT1 |  |  | 4955 | 2.431 | 0.8792 | No |
| 18 | ENSG00000103089 | FA2H |  |  | 5042 | 2.379 | 0.8789 | No |
| 19 | ENSG00000130164 | LDLR |  |  | 5405 | 2.231 | 0.8739 | No |
| 20 | ENSG00000176887 | SOX11 |  |  | 6473 | 1.930 | 0.8568 | No |
| 21 | ENSG00000124920 | MYRF |  |  | 6673 | 1.884 | 0.8544 | No |
| 22 | ENSG00000169247 | SH3TC2 |  |  | 7140 | 1.785 | 0.8475 | No |
| 23 | ENSG00000010278 | CD9 |  |  | 7144 | 1.785 | 0.8483 | No |
| 24 | ENSG00000123384 | LRP1 |  |  | 7159 | 1.782 | 0.8490 | No |
| 25 | ENSG00000172037 | LAMB2 |  |  | 7355 | 1.746 | 0.8466 | No |
| 26 | ENSG00000119718 | EIF2B2 |  |  | 7391 | 1.737 | 0.8469 | No |
| 27 | ENSG00000184144 | CNTN2 |  |  | 7444 | 1.726 | 0.8469 | No |
| 28 | ENSG00000164885 | CDK5 |  |  | 7629 | 1.691 | 0.8446 | No |
| 29 | ENSG00000115738 | ID2 |  |  | 7729 | 1.671 | 0.8438 | No |
| 30 | ENSG00000070785 | EIF2B3 |  |  | 7779 | 1.661 | 0.8438 | No |
| 31 | ENSG00000105329 | TGFB1 |  |  | 8097 | 1.609 | 0.8393 | No |
| 32 | ENSG00000142168 | SOD1 |  |  | 8206 | 1.593 | 0.8383 | No |
| 33 | ENSG00000164690 | SHH |  |  | 8548 | 1.541 | 0.8333 | No |
| 34 | ENSG00000105221 | AKT2 |  |  | 8603 | 1.535 | 0.8332 | No |
| 35 | ENSG00000204305 | AGER |  |  | 8632 | 1.530 | 0.8335 | No |
| 36 | ENSG00000104884 | ERCC2 |  |  | 9389 | 1.432 | 0.8214 | No |
| 37 | ENSG00000145191 | EIF2B5 |  |  | 9494 | 1.418 | 0.8204 | No |
| 38 | ENSG00000115211 | EIF2B4 |  |  | 9817 | 1.381 | 0.8156 | No |
| 39 | ENSG00000177807 | KCNJ10 |  |  | 10396 | 1.318 | 0.8065 | No |
| 40 | ENSG00000100151 | PICK1 |  |  | 10615 | 1.303 | 0.8035 | No |
| 41 | ENSG00000100429 | HDAC10 |  |  | 10661 | 1.298 | 0.8034 | No |
| 42 | ENSG00000173402 | DAG1 |  |  | 11655 | 1.214 | 0.7872 | No |
| 43 | ENSG00000163517 | HDAC11 |  |  | 12176 | 1.167 | 0.7790 | No |
| 44 | ENSG00000111361 | EIF2B1 |  |  | 12244 | 1.160 | 0.7784 | No |
| 45 | ENSG00000184634 | MED12 |  |  | 12781 | 1.118 | 0.7699 | No |
| 46 | ENSG00000172201 | ID4 |  |  | 12983 | 1.103 | 0.7671 | No |
| 47 | ENSG00000254087 | LYN |  |  | 13520 | 1.062 | 0.7586 | No |
| 48 | ENSG00000157933 | SKI |  |  | 13763 | 1.045 | 0.7550 | No |
| 49 | ENSG00000008277 | ADAM22 |  |  | 13969 | 1.029 | 0.7520 | No |
| 50 | ENSG00000131095 | GFAP |  |  | 14245 | 1.007 | 0.7479 | No |
| 51 | ENSG00000128271 | ADORA2A |  |  | 14382 | 0.996 | 0.7461 | No |
| 52 | ENSG00000198947 | DMD |  |  | 14471 | 0.996 | 0.7451 | No |
| 53 | ENSG00000142192 | APP |  |  | 15899 | 0.926 | 0.7214 | No |
| 54 | ENSG00000153902 | LGI4 |  |  | 16174 | 0.908 | 0.7173 | No |
| 55 | ENSG00000184845 | DRD1 |  |  | 16190 | 0.907 | 0.7175 | No |
| 56 | ENSG00000221823 | PPP3R1 |  |  | 16201 | 0.907 | 0.7178 | No |
| 57 | ENSG00000120885 | CLU |  |  | 16210 | 0.906 | 0.7181 | No |
| 58 | ENSG00000171862 | PTEN |  |  | 16769 | 0.871 | 0.7091 | No |
| 59 | ENSG00000242689 | CNTF |  |  | 17037 | 0.855 | 0.7050 | No |
| 60 | ENSG00000196712 | NF1 |  |  | 17466 | 0.829 | 0.6982 | No |
| 61 | ENSG00000148498 | PARD3 |  |  | 17727 | 0.814 | 0.6942 | No |
| 62 | ENSG00000143546 | S100A8 |  |  | 18155 | 0.790 | 0.6874 | No |
| 63 | ENSG00000112414 | ADGRG6 |  |  | 18986 | 0.743 | 0.6737 | No |
| 64 | ENSG00000132970 | WASF3 |  |  | 19002 | 0.742 | 0.6738 | No |
| 65 | ENSG00000133703 | KRAS |  |  | 19512 | 0.715 | 0.6656 | No |
| 66 | ENSG00000185668 | POU3F1 |  |  | 19631 | 0.709 | 0.6639 | No |
| 67 | ENSG00000162576 | MXRA8 |  |  | 20334 | 0.669 | 0.6524 | No |
| 68 | ENSG00000100697 | DICER1 |  |  | 20930 | 0.639 | 0.6426 | No |
| 69 | ENSG00000072415 | MPP5 |  |  | 21258 | 0.622 | 0.6374 | No |
| 70 | ENSG00000166333 | ILK |  |  | 21614 | 0.601 | 0.6317 | No |
| 71 | ENSG00000148180 | GSN |  |  | 21661 | 0.598 | 0.6312 | No |
| 72 | ENSG00000105810 | CDK6 |  |  | 21736 | 0.593 | 0.6303 | No |
| 73 | ENSG00000080815 | PSEN1 |  |  | 22258 | 0.559 | 0.6217 | No |
| 74 | ENSG00000265763 | ZNF488 |  |  | 22606 | 0.537 | 0.6161 | No |
| 75 | ENSG00000124766 | SOX4 |  |  | 22767 | 0.524 | 0.6137 | No |
| 76 | ENSG00000163220 | S100A9 |  |  | 23819 | 0.453 | 0.5961 | No |
| 77 | ENSG00000152784 | PRDM8 |  |  | 24003 | 0.438 | 0.5932 | No |
| 78 | ENSG00000198121 | LPAR1 |  |  | 24490 | 0.399 | 0.5852 | No |
| 79 | ENSG00000146648 | EGFR |  |  | 24583 | 0.392 | 0.5839 | No |
| 80 | ENSG00000184454 | NCMAP |  |  | 24888 | 0.364 | 0.5789 | No |
| 81 | ENSG00000123560 | PLP1 |  |  | 25259 | 0.324 | 0.5728 | No |
| 82 | ENSG00000104419 | NDRG1 |  |  | 25553 | 0.302 | 0.5680 | No |
| 83 | ENSG00000185483 | ROR1 |  |  | 25633 | 0.291 | 0.5668 | No |
| 84 | ENSG00000128602 | SMO |  |  | 26169 | 0.234 | 0.5579 | No |
| 85 | ENSG00000137462 | TLR2 |  |  | 26308 | 0.215 | 0.5556 | No |
| 86 | ENSG00000136869 | TLR4 |  |  | 26317 | 0.214 | 0.5556 | No |
| 87 | ENSG00000134198 | TSPAN2 |  |  | 26909 | 0.129 | 0.5457 | No |
| 88 | ENSG00000104728 | ARHGEF10 |  |  | 29725 | 0.000 | 0.4980 | No |
| 89 | ENSG00000171049 | FPR2 |  |  | 31331 | NaN | 0.4708 | No |
| 90 | ENSG00000148826 | NKX6-2 |  |  | 39041 | NaN | 0.3402 | No |
| 91 | ENSG00000034971 | MYOC |  |  | 43004 | NaN | 0.2731 | No |
| 92 | ENSG00000068903 | SIRT2 |  |  | 52677 | NaN | 0.1093 | No |
| 93 | ENSG00000197921 | HES5 |  |  | 55622 | NaN | 0.0595 | No |
| 94 | ENSG00000198719 | DLL1 |  |  | 56017 | NaN | 0.0528 | No |
Table: GSEA details
[plain text format]

  

Fig 2: GLIAL\_CELL\_DEVELOPMENT(GO:0021782)
  
Blue-Pink O' Gram in the Space of the Analyzed GeneSet

  

Fig 3: GLIAL\_CELL\_DEVELOPMENT(GO:0021782): Random ES distribution
  
Gene set null distribution of ES for
 **GLIAL\_CELL\_DEVELOPMENT(GO:0021782)**
